# Supplementary material for: Entomological surveillance on Aedes aegypti during covid 19 period in Cape Coast, Ghana: Risk of arboviral outbreaks, multiple insecticide resistance and distribution of F1534C, V410L and V1016I kdr mutations
Source: PLoS Negl Trop Dis. 2023 May 31;17(5):e0011397. doi: 10.1371/journal.pntd.0011397 (PMC10259782; doi:10.1371/journal.pntd.0011397)
Supplement: Attachment — Submitted filename: cover letter_Plos NTD R2.pdf [file pntd.0011397.s003.pdf]

Department of Conservation Biology and Entomology
School of Biological Sciences
University of Cape Coast,
Ghana

18th March 2023

PLOS Neglected Tropical Diseases
San Francisco, California.

Dear Editors in Chief,

I will like to submit the revised manuscript entitled "Entomological surveillance on *Aedes aegypti* during covid 19 period in Cape Coast, Ghana: risk of arboviral outbreaks, multiple insecticide resistance and distribution of F1534C, V410L and V1016I kdr mutations". We are very grateful to the up-building comments and suggestions from the members of the editorial board and the independent reviewers. The manuscript has been modified according to the reviewers' recommendation.

Thank you.

Yours faithfully,

adus

Andreas Kudom

Response to reviewer's

Reviewer #1:

The authors have adequately addressed most comments raised in the first round. The only recommendation would be to discuss the lack of monitoring for the infectious status of mosquitoes as a limitation of the study.

Response: The infectious status of mosquitoes as a limitation of the study has been discussed (line 314-317).
