# Supplementary material for: Entomological surveillance on Aedes aegypti during covid 19 period in Cape Coast, Ghana: Risk of arboviral outbreaks, multiple insecticide resistance and distribution of F1534C, V410L and V1016I kdr mutations
Source: PLoS Negl Trop Dis. 2023 May 31;17(5):e0011397. doi: 10.1371/journal.pntd.0011397 (PMC10259782; doi:10.1371/journal.pntd.0011397)
Supplement: Attachment — Submitted filename: response to reviewers comment_2.pdf [file pntd.0011397.s004.pdf]

Response to Reviewer's Responses to Questions

Reviewer #1: The authors have adequately addressed most comments raised in the first round. The only recommendation would be to discuss the lack of monitoring for the infectious status of mosquitoes as a limitation of the study.

Response: The infectious status of mosquitoes has been acknowledge as a limitation of the study and the manuscript has been modify accordingly (line 314-320).
