# Supplementary material for: Entomological surveillance on Aedes aegypti during covid 19 period in Cape Coast, Ghana: Risk of arboviral outbreaks, multiple insecticide resistance and distribution of F1534C, V410L and V1016I kdr mutations
Source: PLoS Negl Trop Dis. 2023 May 31;17(5):e0011397. doi: 10.1371/journal.pntd.0011397 (PMC10259782; doi:10.1371/journal.pntd.0011397)
Supplement: Attachment — Submitted filename: Response to reviewers_PLOS NTD.docx [file pntd.0011397.s002.docx]

**Response to Reviewer's Questions**

Reviewer #1:

1.      It is mentioned lLine 256) that the study assessed the risk of transmission of Aedes-borne diseases. This is not true, but should have been performed (infection status- DENV/CHIKV etc of mosquitoes) to be honest as this is most relevant.

Response: We agree with you that the direct assessment should have been screening for the virus in the mosquitoes. However, WHO also recommend the use of entomological parameters such as the Laval indices used in this study as an indirect way of assessing the risk of transmission of *Aedes* borne diseases. In future study, we hope to be able to screen for the virus in the mosquito.

2.      Metabolic resistance, a major mechanism of Aedes aegypti resistance, was not studies neither at the phenotypic nor at the molecular levels. For example, the OP/carbamate resistance can not be attributed to kdr mutations.

Additional comments

Response: We agree that the kdr detected in this cannot explain fully the resistance observed in this study. We have indicated the limitation accordingly (Line 400- 409).

3.      The clean-up exercises, as they are mentioned, should be described in more, technical details, and discussed more elaborately to show how they could have helped the situation.

Response: the clean-up exercise was the usual sweeping and gathering the rubbish from the community. Market places in the community were fumigated. However, for this exercise cleaning included areas that are normally left out during normal cleaning by the residents and the rubbish or refuse was properly managed.

4.      Rows 41-43, conclusions: this is not supported by the study’s data and is dubious to say the least; insecticides remain the mainstay of vector control.

Response: Insecticides remain the mainstay of vector control. The sentence was trying to suggest an alternative tools to support the insecticide-based tools due to the emergence of insecticide. The sentence has been modified to reflect this meaning (Line 37-40).

5.      Introduction: This section would benefit from information regarding insecticide resistance and its mechanisms, at least for target site that was studied.

Response: Additional information has been added in that respect (Line 95-114)

6.      At attempt should be made to explain the differences in insecticide resistance (phenotypic and molecular) at the different sites studied.

Response: the paragraph has been modified to incorporate your suggestions (Line 353-400).

7.      Line 306: Ae. aegypti is mentioned, but in Table 1, only Aedes genus is presented. Where mosquitoes identified to species level?

Response: Yes, Aedes mosquitoes were identified to the species level. However, the rest of the mosquitoes were identified to the genus level.

Reviewer #2:

Line 28 -> change "to adults were tested against four insecticides; " to "to adults were tested against four insecticides: "

Response: the sentence has been modified

Would it be possible to obtain precipitation data from 2017 and 2020? Your data show a lower outbreak risk. Could it be due to lower precipitation and not directly associated with Covid measures? Please add anything indicating other potential factors regarding the lower levels in 2020 compared to 2017.

Response: Precipitation data has been added and discussed accordingly (Line 154 (Figure 1, 300-307)

Table 1 shows that you collected a lot of larvae from concrete tanks (1,088 larvae), representing almost half of your total larvae collected in HWSC. Did you consider that your insecticide resistance data might is biased toward this group of larvae since you used F1s? How big were these tanks? Could it be that the larvae you collected from there were all related to one single female that laid eggs there? One could easily remove water bottles or smaller plastic containers during clean-ups during the Covid measures, but the large water reservoir would be impossible.

Response: The larvae were collected in cement tanks, which were found in various houses. It is one of the main water storage containers in the community. Most water storage containers including the cement tanks were found inside the houses.

Would it be possible to add epidemiological data from 2017 and 2022 for Dengue, for example? The study's conclusions rely on lower indexes, but it would be great if the number of Dengue cases also decreased. I guess that people were not moving around too much, reducing the transmission of Dengue.

Response: the country does not have epidemiological data on dengue. This is one of the reasons why we are using entomological data to assess the risk.

Figure 4 shows the compound genotype frequencies of each mutation. How did you "phase" them? Since you have heterozygous genotypes, it becomes impossible to determine which mutation is on each parental chromosome. For example, three loci can have up to eight three possible haplotypes.

Response: Please, I do not fully understand the question. However, the three mutations were screened in individual mosquitoes. Some were homozygous to the mutation or heterozygous as well as the wild type for each of the mutation. Then, we enumerated all the possible genotype combination of the three mutations.

Reviewer #3:

1. Rewrite the title to make it better.

Response: the title has been modified to “Entomological surveillance on Aedes aegypti during covid 19 period in Cape Coast, Ghana: risk of arboviral outbreaks, multiple insecticide resistance and distribution of F1534C, V410L and V1016I kdr mutations”.

2.      Your response “All relevant data are within the manuscript” to the question “Describe where the data may be found in full sentences……” is not sufficient. Provide your databases as supplementary files or links to where to access it.

Response: All the household and insecticide resistance data were used for the analysis (Table 1, Fig 2), (Fig3-4). The genotype frequencies of the three *kdr* mutations has been attached as a supplementary file.

4.      This research study was conducted from July to September 2020 when COVID-19 infections were high and movement restrictions in place. How did you obtain permission to conduct this study and what precautions did you put in place to prevent contributing towards COVID-19 transmissions?

Response: there was an ongoing mosquito collection in the community before the pandemic. Unlike Accra, as described in the Materials and Method section, movement in Cape Coast was not banned. All the Covid 19 protocols, particularly with wearing of mask were observed and the community leaders led as to each house.

5.      Page 12 Lines 130-131, clearly describe how the pre-adult mosquito stages were sampled from the selected houses.

Response: A dipper and pipette were used to collect the larvae. For containers that had more than 20litres of water, pupae were collected by filtering the water with a mesh sieve. Pupae from containers containing fewer than 20litres of water were picked using a pipette and those from very small containers were emptied into trays.

6.      Page 12 Lines 134-135, provide a brief description of House index (HI), Container index (CI) and Breteau index (BI) like HI was expressed as the percentage of houses infested with Aedes larvae; CI as the percentage of containers infested; and BI as the number of positive containers per 100 inspected houses.

Response: it has been added (line 156-159)

7.      Did the house owners provide consent to sample their houses?

Response: Yes. Verbal consent was given by each head of the household. We did not work in houses that the head was absent or did not give us the consent.

8.      Did this research study obtain ethical approval? If yes, indicate the approval number and the Institutional Review Board (IRB) or Ethical Review Committee (ERC) that approved it.

Response: No. In 2017 the institutional review board wave the ethical approval for the work since we were only dealing with mosquitoes without human or animal involvement. Mosquito collection has been ongoing since 2017 up till now.

9.      Data analysis section is mission. Indicate how you managed and analyzed the data you collected.

Response: data analysis has been added to each sub section under the materials and methods (156-159, 171-172, 194-196)

10.     Indicate how the spatial distribution map in Figure 1 was made.

Response: Coordinates from each house was taken and Arch GIS was used to generate the map. Then, the map was imported into PowerPoint, where pictures of water holding containers were added to it.

11.     Read the PLOS NTD Instructions to Authors on Figures and Tables and comply appropriately.

Response: Thank you. We have done it.

12.     Omit sub-titles in the discussion section.

Response: they have been removed. Thank you

13.     Did you ask how long water was stored in each of the containers before it was fully emptied?

Response: No. Usually, the big tanks are not completely emptied. They continue to top them up as they use.

14.     Page 22 Lines 258-259, cite the previous study that was done in 2017 and provide a summary of its findings.

Response: A summary from the previous study has been provided (Line 284-290)

15.     With clear understanding of the ecology and behavior of Aedes aegypti, clearly discuss how important the multiple kdr mutations are to it.

Response: the discussion has been modified.

16.     In your discussion, find a goof link among multiple kdr mutations, risk of Ae. aegypti arboviral outbreaks and COVID-19 restrictions.

Response: based on your suggestion, the discussion has been modified accordingly.

17.     Discuss the limitations of this study.

Response: the major limitation was the small sample size, which has been discussed (line 310-314) as well as the limited role of kdr mutation in explaining the resistance found in this study (line: 400-409).

18.     Make your conclusion brief and clear.

Response: the conclusion has been modified to make it brief and concise.
